# Supplementary figures and images for: Genomic data from NSCLC tumors reveals correlation between SHP-2 activity and PD-L1 expression and suggests synergy in combining SHP-2 and PD-1/PD-L1 inhibitors
Source: PLoS One. 2021 Aug 26;16(8):e0256416. doi: 10.1371/journal.pone.0256416 (PMC8389511; doi:10.1371/journal.pone.0256416)

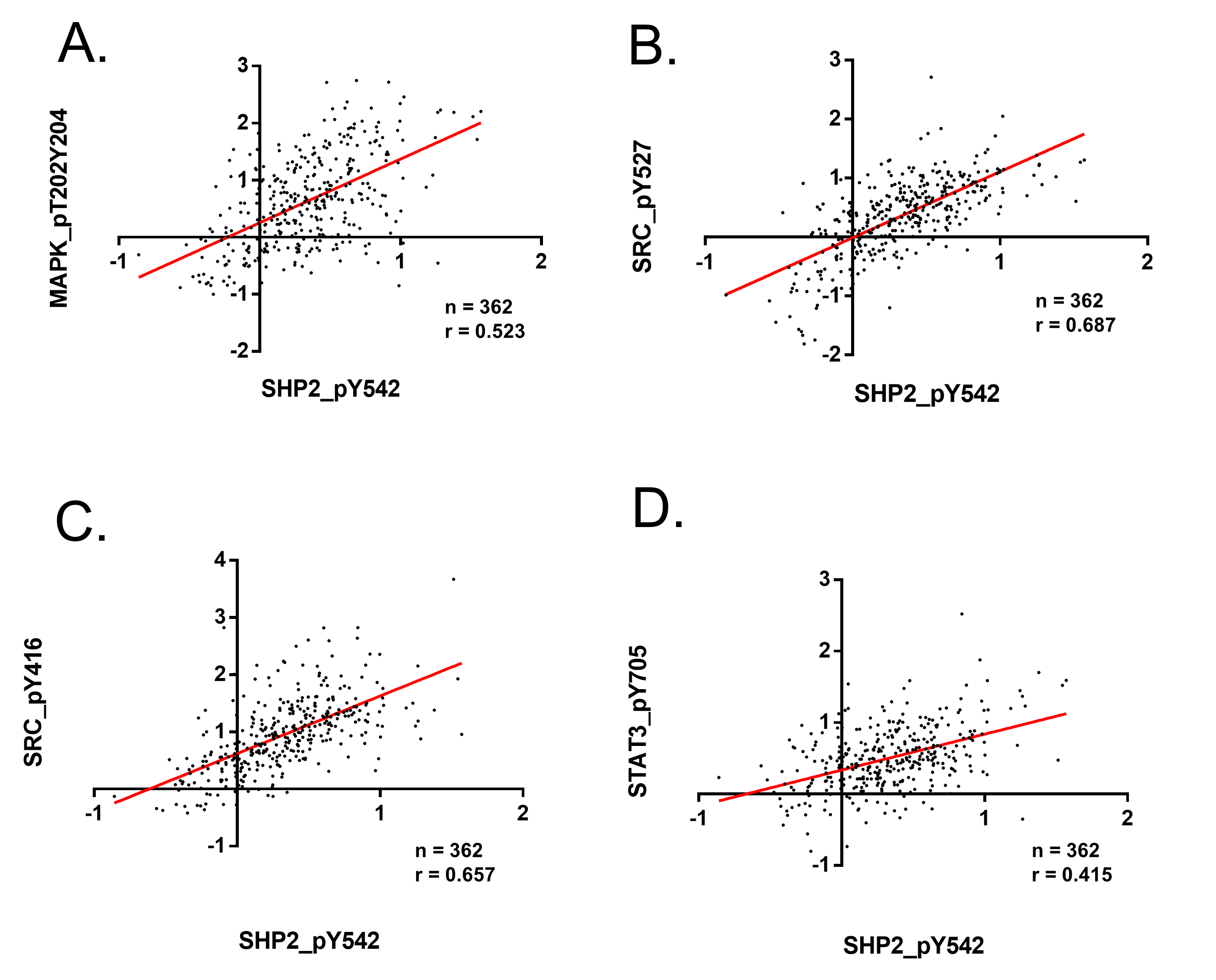

Supplement: S1 Fig — A. Two-tailed non-parametric Spearman correlation analysis of RPPA protein expression data for Y542 phosphorylated SHP-2 and T202/Y204 phosphorylated MAPK B. Y527 phosphorylated Src kinase C. Y416 phosphorylated Src kinase D. Y705 phosphorylated STAT3 from 362 adenocarcinomas taken from The Cancer Proteome Atlas (TCPA: https://gdc.cancer.gov/about-data/publications/pancanatlas) LUAD-L4 dataset. The red line represents a linear regression line of best fit. (TIF) [file pone.0256416.s001.tif]

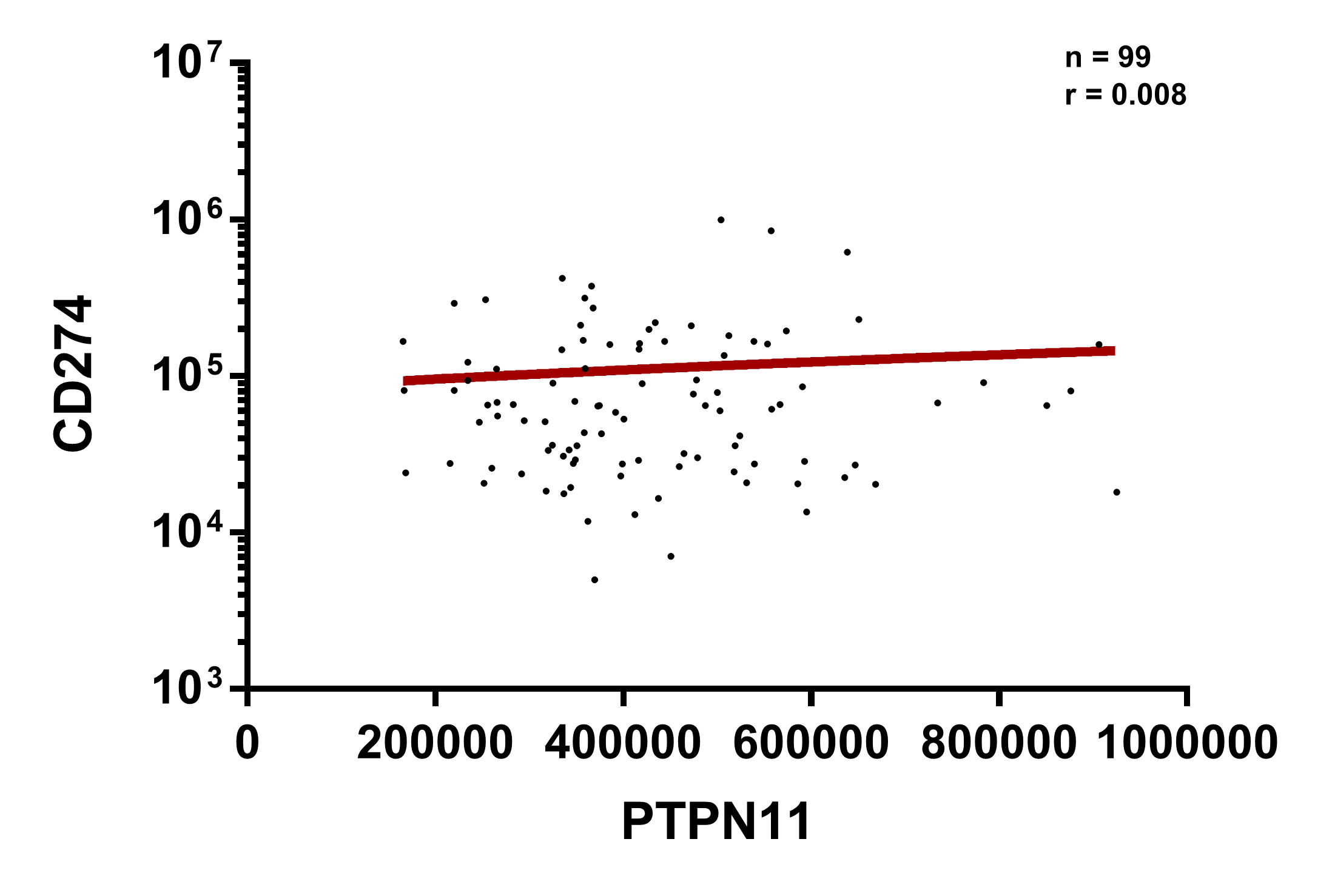

Supplement: S2 Fig — Two-tailed non-parametric Spearman correlation analysis of bulk RNA-seq FPKM-UQ values taken from TCGA (GDC: https://gdc.cancer.gov/about-data/publications/pancanatlas) for 99 patients harboring mutations in the KRAS gene found in the TCGA-LUAD dataset. The red line represents a linear regression line of best fit. (TIF) [file pone.0256416.s002.tif]

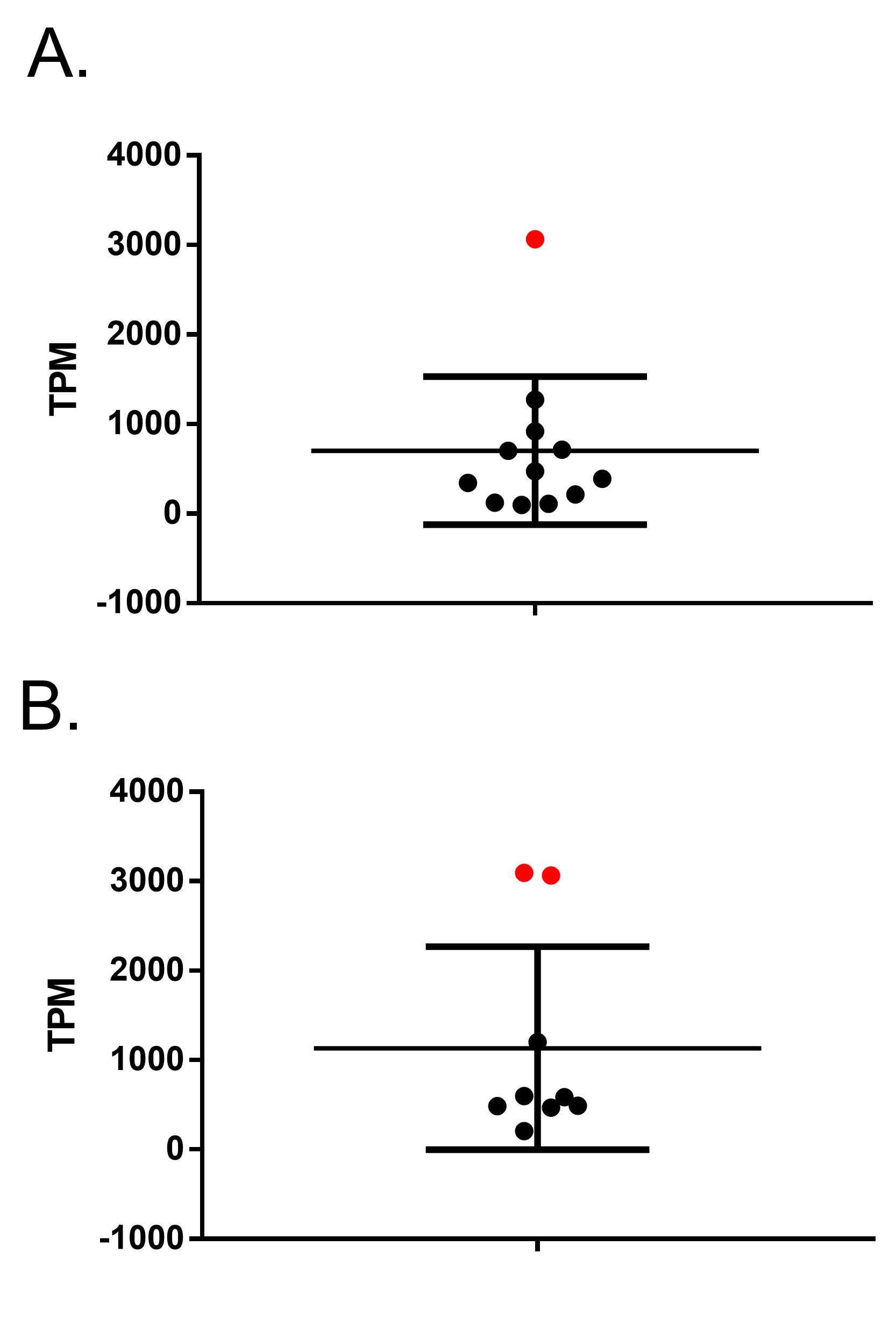

Supplement: S3 Fig — Box and whisker plot of PTPN11 TPM values for NSCLC patients who did not respond to ICI therapy (A) or patients who did respond (B). Outliers, highlighted in red, were determined by the 1.5 interquartile range (IQR) method which adds 1.5 times the IQR to the third quartile and excludes data points that fall above that value, and subtracts 1.5 times the IQR from the first quartile and excludes data points that fall below that value. (TIF) [file pone.0256416.s003.tif]
