# Supplementary material for: Genomic data from NSCLC tumors reveals correlation between SHP-2 activity and PD-L1 expression and suggests synergy in combining SHP-2 and PD-1/PD-L1 inhibitors
Source: PLoS One. 2021 Aug 26;16(8):e0256416. doi: 10.1371/journal.pone.0256416 (PMC8389511; doi:10.1371/journal.pone.0256416)
Supplement: Attachment — Submitted filename: Response to Reviewers.pdf [file pone.0256416.s004.pdf]

UNIVERSITY OF KENTUCKY

Esther P. Black, PhD
College of Pharmacy
Department of Pharmaceutical Sciences
343 TODD Building
Lexington, KY 40536-0596
859 323-5898
fax 859 257-7564

July 22, 2021

We thank each reviewer and editor for comments that have improved the manuscript. We have added an author who provided the automation of data capture an analysis. Importantly, we attempted to acquire another data set of 80+ NSCLC patients with RNA seq and ICI response data from Genentech. The process is ongoing, and we are likely not going to be able to use these data in a publication for the foreseeable future regardless of release of those data.
Responses to the reviewers are below in green font.

Reviewer #1 comments:
The manuscript, “Real world genomic data supports combined use of SHP-2 and PD-1/PD-L1 inhibitors in solid tumors” aims at establishing a relationship between SHP-2 and PD-1/PD-L1 using available patient genomic and proteome from data base. The authors used statistical analysis to draw information from the existing data set and made a good attempt to test their hypothesis that inhibition of SHP-2 will improve the activity of ICI inhibitors that target PD-1 or PD-L1 in lung cancers. However, their inference of the data are extensions drawn based on inconclusive data.

Language has been added to both the Methods and Results and the Discussion sections to be more faithful to the interpretations of the data.

In order to draw a correlation between SHP-2 and PD-L1, the authors analyzed TCPA data. As SHP-2 exists in active (phosphorylated) and inactive (unphosphorylated) form, it is important to analyze the levels of PD-L1 with reference to both SHP-2 forms. However, TCPA data set is missing a crucial control of inactive SHP-2. In the absence of this information, authors could have used another reference biomarker associated with SHP-2 signaling as an internal control. The figure 1 data does not establish a strong relationship between SHP-2 and PD-1 as the authors predicted/claimed.

While we were unable to measure unphosphorylated SHP-2 as an internal control, we identified 3 proteins involved in signaling pathways which SHP-2 activity is known to positively regulate. Phosphorylated forms of these proteins (Src, STAT3, and MAPK) are indicative of pathway activation, and we can use these correlations to validate the impact of pY542-SHP2 on SHP2 activity.
We also showed a positive correlation in mRNA levels between PTPN11 and CD274, supporting the notion that SHP-2 and PD-L1 proteins are co-expressed in NSCLC tissue. However, this correlation was only found when analyzing the full TCGA—LUAD

dataset not the TCGA-LUAD-L4 subset. To extend these findings, we identified another dataset in cBioPortal to investigate the relationship between SHP-2 and PD-L1 expression. This dataset contained mRNA expression data from a study in 2020 (Chen et al.) that contained a larger sample size (n=169) of LUAD tumors. Analysis of these data uncovered a similar trend in that SHP-2 and PD-L1 mRNA levels are positively correlated.

The authors then analyzed data from melanoma tumors to support their hypothesis related to lung cancers. The two tumors possess their unique biomarkers and are different from each other in several ways to form comparable groups for analysis. The data other than Figure 1A, establishes that there is no association between expression of PTPN11 and CD274 on the basis of mRNA levels. However, the authors extrapolated this to draw conclusion that it is SHP-2 activity and not the expression levels of SHP-2 that impacts PD-L1 expression. As stated before, in the absence of data for inactive SHP-2, there is no direct evidence to support this statement.

Language has been added to the end of Methods and Results section 1 to temper our assertion that SHP-2 activity, not expression, impacts PD-L1 expression.

Additionally, sample size in Figure 2B and Figure 2C are very low to analyze the effect SHP-2 and PD-1/PD-L1 on ICI inhibitors.

This is true, so language has been added in the Discussion section to clarify that because the data was compiled from smaller studies, we are unable to confirm that SHP-2 and PD-L1 inhibition would synergize. From our findings from the TCPA/TCGA (https://gdc.cancer.gov/about-data/publications/pancanatlas) and cBioPortal (Chen J et al. 2020), we assert that inhibiting SHP-2 would result in increased levels of tumoral PD-L1 protein that would result in an increased likelihood of response to ICI therapy. In NSCLC, tumoral expression of PD-L1 >50% is linked to better response rates as uncovered by KEYNOTE trials 10, 24, and 25.

Finally, there is no direct and clear evidence in the manuscript to support the title of this manuscript “Real world genomic data supports combined use of SHP-2 and PD-1/PD-L1 inhibitors in solid tumors”. The data from none of the figures or tables listed in the manuscript back this claim.

The title of the manuscript has been changed to “**Genomic data from NSCLC tumors reveals correlation between SHP-2 activity and PD-L1 expression and suggests synergy in combining SHP-2 and PD-1/PD-L1 inhibitors**”

Reviewer #2 comments:
I read with interest this paper and given the limitations reported by the authors in terms of response data and trial results, the strategy they followed is correct, but preliminary in impact.

With wider study assessment would become more robust, always as a preliminary study. I wonder whether this weakness can be solved or otherwise (as per the current Title) the real-world data support of combined SHP-2 : PD-1/PD-L1 inhibitors use in solid tumors should be scaled a bit down.
Please see the responses to Reviewer #1 as we believe these address each of your concerns also.

Editor comments:

1. Please ensure that your manuscript meets PLOS ONE's style requirements, including those for file naming. The PLOS ONE style templates can be found at

https://journals.plos.org/plosone/s/file?id=wjVg/PLOSOne_formatting_sample_main_body.pdf and

https://journals.plos.org/plosone/s/file?id=ba62/PLOSOne_formatting_sample_title_authors_affiliations.pdf

We believe each file name adheres to your naming standards.

2. Please provide additional details regarding participant consent. In the ethics statement in the Methods and online submission information, please ensure that you have specified (1) whether consent was informed and (2) what type you obtained (for instance, written or verbal, and if verbal, how it was documented and witnessed). If your study included minors, state whether you obtained consent from parents or guardians. If the need for consent was waived by the ethics committee, please include this information.Once you have amended this/these statement(s) in the Methods section of the manuscript, please add the same text to the “Ethics Statement” field of the submission form (via “Edit Submission”).

For additional information about PLOS ONE ethical requirements for human subjects research, please refer to http://journals.plos.org/plosone/s/submission-guidelines#loc-human-subjects-research.

We are not the authors of any of the study data we used. Thus, we defer to the assertion by each group of authors (Jerby-Arnon et al. 2018, Hwang S et al. 2020, Chen J et al. 2020) that appropriate consent was gathered.

3. We note that you have included the phrase “data not shown” in your manuscript. Unfortunately, this does not meet our data sharing requirements. PLOS does not permit references to inaccessible data. We require that authors provide all relevant data within the paper, Supporting Information files, or in an acceptable, public repository. Please add a citation to support this phrase or upload the data that corresponds with these findings to a stable repository (such as Figshare or Dryad) and provide and URLs, DOIs, or accession numbers that may be used to access these data. Or, if the data are not a core part of the research being presented in your study, we ask that you remove the phrase that refers to these data.

We removed the “data not shown” phrase and included the origin of all the data from public repositories and primary manuscripts.

Sincerely,

Penni Black, Ph.D.
Associate Professor and Director of Professional Studies
